# Assessment of submicroscopic infections and gametocyte carriage of *Plasmodium falciparum* during peak malaria transmission season in a community-based cross-sectional survey in western Kenya, 2012

**DOI:** 10.1186/s12936-016-1482-4

**Published:** 2016-08-19

**Authors:** Zhiyong Zhou, Rebecca M. Mitchell, Simon Kariuki, Christopher Odero, Peter Otieno, Kephas Otieno, Philip Onyona, Vincent Were, Ryan E. Wiegand, John E. Gimnig, Edward D. Walker, Meghna Desai, Ya Ping Shi

**Affiliations:** 1 Malaria Branch, Division of Parasitic Diseases and Malaria, Center for Global Health, Centers for Disease Control and Prevention, Atlanta, GA USA; 2 Centre for Global Health Research, Kenya Medical Research Institute, Kisumu, Kenya; 3 Department of Microbiology and Molecular Genetics, Michigan State University, East Lansing, MI USA

**Keywords:** *Plasmodium falciparum*, Gametocytes, Risk factors, Antimalarials, ITNs, Kenya

## Abstract

**Background:**

Although malaria control intervention has greatly decreased malaria morbidity and mortality in many African countries, further decline in parasite prevalence has stagnated in western Kenya. In order to assess if malaria transmission reservoir is associated with this stagnation, submicroscopic infection and gametocyte carriage was estimated. Risk factors and associations between malaria control interventions and gametocyte carriage were further investigated in this study.

**Methods:**

A total of 996 dried blood spot samples were used from two strata, all smear-positives (516 samples) and randomly selected smear-negatives (480 samples), from a community cross-sectional survey conducted at peak transmission season in 2012 in Siaya County, western Kenya. *Plasmodium falciparum* parasite presence and density were determined by stained blood smear and by 18S mRNA transcripts using nucleic acid sequence-based amplification assay (NASBA), gametocyte presence and density were determined by blood smear and by Pfs25 mRNA-NASBA, and gametocyte diversity by Pfg377 mRNA RT-PCR and RT-qPCR.

**Results:**

Of the randomly selected smear-negative samples, 69.6 % (334/480) were positive by 18S-NASBA while 18S-NASBA detected 99.6 % (514/516) smear positive samples. Overall, 80.2 % of the weighted population was parasite positive by 18S-NASBA vs 30.6 % by smear diagnosis and 44.0 % of the weighted population was gametocyte positive by Pfs25-NASBA vs 2.6 % by smear diagnosis. Children 5–15 years old were more likely to be parasitaemic and gametocytaemic by NASBA than individuals >15 years old or children <5 years old while gametocyte density decreased with age. Anaemia and self-reported fever within the past 24 h were associated with increased odds of gametocytaemia. Fever was also positively associated with parasite density, but not with gametocyte density. Anti-malarial use within the past 2 weeks decreased the odds of gametocytaemia, but not the odds of parasitaemia. In contrast, recent anti-malarial use was associated with lowered parasite density, but not the gametocyte density. Use of ITNs was associated with lower odds for parasitaemia in part of the study area with a longer history of ITN interventions. In the same part of study area, the odds of having multiple gametocyte alleles were also lower in individuals using ITNs than in those not using ITNs and parasite density was positively associated with gametocyte diversity.

**Conclusion:**

A large proportion of submicroscopic parasites and gametocytes in western Kenya might contribute to the stagnation in malaria prevalence, suggesting that additional interventions targeting the infectious reservoir are needed. As school aged children and persons with anaemia and fever were major sources for gametocyte reservoir, these groups should be targeted for intervention and prevention to reduce malaria transmission. Anti-malarial use was associated with lower parasite density and odds of gametocytaemia, but not the gametocyte density, indicating a limitation of anti-malarial impact on the transmission reservoir. ITN use had a protective role against parasitaemia and gametocyte diversity in western Kenya.

**Electronic supplementary material:**

The online version of this article (doi:10.1186/s12936-016-1482-4) contains supplementary material, which is available to authorized users.

## Background

Scale-up of malaria control interventions in endemic countries has resulted in drastic declines in malaria morbidity and all-cause mortality in many African countries. In western Kenya, after decades of malaria prevention and treatment measures, such as insecticide-treated nets (ITNs), intermittent preventive treatment in pregnancy (IPTp) and artemisinin-based combination therapy (ACT), the prevalence of *Plasmodium falciparum* in children below 5 years of age as diagnosed by blood smears declined from 60 % in 2003 to 26 % in 2008, but rose to 41 % in 2009 [1]. Malaria prevalence was 38 % in children <5 years in 2010 [2]. It is unclear if this reversal and stagnation in malaria prevalence in this area is associated with submicroscopic infections and the sexual stage reservoir, gametocytes.

Malaria transmission relies on sexual stage parasites, the gametocytes that derive from a small fraction of asexual parasites. Immature forms of *P. falciparum* gametocytes (stage I–IV) are sequestered in organ tissue, mainly in the bone marrow [3]. The gametocyte maturation process in the bone marrow requires 10–12 days [3, 4], and mature gametocytes then release into peripheral blood and persist with an average circulation time of 4.6–6.5 days [5–7] using microscopy. An average duration of gametocytaemia has been reported at 55 days (95 % CI 28.7–107.7) using molecular methods following non-ACT drug treatment [6]. The mature gametocytes (stage V) which are responsible for parasite transmission from human to mosquitoes often circulate at low densities [8]. In this context, sensitive molecular detection tools could improve detection of low densities of gametocytes and submicroscopic infections to identify potential transmission reservoir. RNA-based detection methods, such as real-time quantitative nucleic acid sequence based amplification (NASBA) technology, are widely used and highly sensitive with a quantitative detection limit of about 20 parasites/ml blood for research purposes [9]. The Pfs25-NASBA can detect 3- to 10-fold more gametocytes than microscopy [10]. It has been shown by the highly sensitive molecular methods that the gametocyte reservoir is much larger than previously detected or reported [8, 10].

Gametocyte production and epidemiology could be associated or influenced by several factors that include transmission intensity, exposure to interventions like ITNs and anti-malarial drugs, host age/immunity, asexual parasite density, anaemia, multiplicity of infection [4, 11–15]. In high malaria transmission areas, gametocyte carriage is most prevalent in children under 5 years of age [4] and declines with increasing age in parallel with asexual parasite prevalence and densities due to increased host anti-parasite immunity [16, 17]. In areas of low transmission intensity, gametocyte prevalence is low among all age groups [18] and the density of gametocytes relative to that of asexual parasites increases with age [4, 19]. Studies have reported a positive association between gametocyte density and the proportion of infected mosquitoes [20]. However, some reports have shown that mosquito infection is not directly proportional to the density of gametocytes in human blood and submicroscopic gametocytes could also infect mosquitoes and sustain malaria transmission [4, 10, 20–22].

Anti-malarials are also found to affect malaria transmission [4, 15, 23, 24]. Artemisinin derivatives are very effective in clearing asexual parasites and reduce immature gametocytes, but may not affect mature gametocytes [25, 26]. Artemether-lumefantrine (AL), was officially introduced in Kenya in 2004 as the first-line drug for treatment of uncomplicated malaria but extensively implemented in 2006 including western Kenya [27]. In western Kenya, a large proportion of asymptomatic infections are associated with submicroscopic parasite densities [28]. A cohort study conducted in rural Kisumu County in western Kenya has reported that treatment of asymptomatic parasitaemic individuals with AL reduced the proportion of individuals who became gametocytaemic during the first 12 weeks of follow-up [29]. However, other studies showed that residual submicroscopic gametocytes after treatment with ACT occurred commonly in Kenya and was associated with a longer duration of gametocyte carriage and a higher transmission potential [7, 28].

ITNs have been deployed for over 16 years and remain the primary malaria intervention in western Kenya. ITNs significantly reduce malaria morbidity and all-cause mortality in children less than 5 years old [30, 31]. ITNs also suppress the mosquito populations and reduce their ability to transmit malaria by 70–90 % [32]. An earlier study has shown that multi-clone *P. falciparum* gametocytes can persist three times longer than those from single-clone infections [33]. Multiple clone infections may increase the male clones in female-biased sex ratio of gametocytes [4, 34] and clones with a male-biased ratio were more infectious to mosquitoes in vitro [34, 35]. However, although ITNs have proved to be an efficacious and cost-effective vector control intervention to reduce clinical disease and malaria transmission, their long term impact on gametocyte carriage and gametocyte diversity is unknown.

Given the complex biology and epidemiology of gametocytes that could be influenced by multiple factors including ITN use and anti-malarial treatment, the objectives of this study were: (1) to estimate the level of submicroscopic infection and gametocyte carriage in circulating blood, measured by molecular tools, in a region of western Kenya, where malaria transmission has stagnated since 2009, (2) to assess the risk factors associated with gametocytaemia, and (3) to determine the associations between malaria control interventions and gametocyte carriage. Results from this study could be useful in understanding and improving the interventions on transmission reservoirs and in providing the information for development of new strategies for transmission reduction and elimination of malaria.

## Methods

### Study area and population

This study was part of a community-based annual cross sectional survey to evaluate impact of malaria control interventions on malaria parasitaemia and anaemia within the KEMRI/CDC Health and Demographic Surveillance System (HDSS) [36]. The cross sectional survey was conducted during peak malaria transmission season from June to July of 2012 in the two adjacent areas of Asembo (Rarieda district) and Karemo (Siaya district), Nyanza region of western Kenya. Use of ITNs has been consistently high in the Asembo area since 1997 while ITNs were introduced in Karemo in 2004 and scaled up in 2006. The entomological inoculation rate (EIR) estimated by pyrethrum spray catches has been consistently at <10 infectious bites per person per year since 2008 (CDC, unpublished data) although recent estimates of EIR through human landing catch indicate the pyrethrum spray catches may underestimate EIRs [37].

### Survey data collection and sample selection

The sampling frame for year 2012 survey included all households in the study areas with at least one child under 5 years old. From this sampling frame, households were selected randomly via probability sampling and all individuals above 1 month of age were sampled in each selected household. A total of 1779 samples was collected for this survey. Self-reported information on age, fever, ITN and recent anti-malarial use were collected based on the study questionnaire. Participants were categorized into three age groups (<5 years old, 5–15 years old, and >15 years old). Fever was defined as self-reported fever within the past 24 h. ITN use was defined as having slept under an ITN the night prior to the survey. Recent anti-malarial (AM) use was defined as receiving anti-malarials (96 % had taken AL) in any dosage at any time within the 2 weeks prior to the survey. During the survey, finger prick blood sample was collected to prepare blood smears to determine the presence of parasitaemia and gametocytaemia by microscopy [38]. Haemoglobin level was determined using portable HemoCue photometers (HemoCue AB, Angelholm, Sweden).

In addition, 50 µL of whole blood was collected into each spot of a Whatman 903 filter paper and dried overnight at room temperature. The dried blood spot (DBS) was sealed tightly with desiccants and a moisture indicator, and shipped to the CDC laboratory in Atlanta, USA within 2 months after sample collection and stored at −80 °C until use.

For the present study, a total of 999 DBS samples from 1779 samples collected during the survey were tested to determine submicroscopic infection and gametocyte carriage. Among them, 446 samples were from Asembo, including all 221 smear-positive and a random selection of 225 from 600 smear-negative samples, and 553 samples were from Karemo, including 298 smear-positive and 255 from 660 randomly selected smear-negative samples. Random sampling of the smear negative samples was performed by Random Sample Excel Professional plus 2010 in Excel, based on the sample size calculated by the program online [39].

### Laboratory tests

#### Nucleic acid extraction

Total RNA was extracted from DBS samples using QIAshredder and RNeasy mini kits (QIAGEN, Valencia, CA) according to the QIAGEN protocols. A whole spot from a DBS sample was cut and used for RNA extraction. After RNA extraction, DNase digestion on one of three aliquots of RNA sample was performed with Ambion DNA-free DNA removal kits (Thermo Fisher Scientific, Waltham, MA) for use in Pfg377 reverse transcription polymerase chain reaction (RT-PCR) and RT-qPCR assays. The other two RNA aliquots without Ambion DNA-free kit treatment were used for 18S-NASBA and Pfs25-NASBA assays.

#### Total parasite load detected by 18S-NASBA

The mRNA transcripts of 18S small subunit rRNA gene of *P. falciparum* were measured for detection of malaria infections and submicroscopic parasites. The primers and molecular beacon probes of 18S-NASBA used were based on previously published methods [9]. The assay was performed on EasyQ analyser (BioMerieux, Durham, NC) using the Nuclisens Basic Kit in a total reaction volume of 10 µL per reaction at 41 °C for 90 min. Positivity was calculated with the time-to-positivity based on the time-point of amplification at which the fluorescence passed a given threshold (above the mean fluorescence of three negative controls plus 20SD) as described by Schneider et al. [9]. In order to quantify the parasite density in samples, a standard curve was made in duplicate by 10-fold dilution series (10^4^–10^−2^ parasites/µL of blood) from cultured 3D7 ring stage parasites. The limit of quantification (LOQ) for 18S-NASBA was 0.01 parasites/µL blood based on the standard curve established in this laboratory.

#### Mature stage V gametocyte detected by Pfs25-NASBA

Pfs25 mRNA was used to detect stage V gametocytes circulating in the host blood and Pfs25-NASBA was performed as previously described [10, 40, 41]. The Pfs25-NASBA assay was carried out in a volume of 10 µL per reaction and 2.5 µL of isolated RNA was used in each reaction. Positivity was calculated with the time-to-positivity at the fluorescence passed a given threshold, the mean fluorescence of three negative controls plus 20SD [10]. In order to measure the gametocyte density, a standard curve was made in duplicate by 10-fold serial diluted 3D7 stage V gametocytes (from 1.8 × 10^4^–1.8 × 10^−2^ gametocytes/µL) in culture blood (Johns Hopkins University, Malaria Research Institute, Baltimore MD). The limit of quantification (LOQ) for Pfs25-NASBA was 0.018 gametocytes/µL based on the standard curve established in this laboratory.

#### Gametocyte diversity assessed by Pfg377 RT-PCR and RT-qPCR

For detection of gametocyte diversity, Pfg377 reverse transcription polymerase chain reaction (RT-PCR) was performed according to a published protocol [42]. The Pfg377 mRNA are only expressed in female gametocytes from stage III onward [43, 44] and the Pfg377 gene contains four regions of repetitive sequence, the most polymorphic being region 3. This assay was designed based on the most polymorphic region 3, which encodes seven degenerate amino acid repeats (21 base pairs) for identification of multiple gametocyte clones within host [43]. Pfg377 RT-PCR was performed using SuperScript III one-step RT-PCR system with platinum Taq RNA polymerase (Thermo Fisher Scientific, Waltham MA). Concurrently, a conventional DNA PCR was performed using the same RNA sample to rule out contamination with genomic DNA. RT-PCR products were run on a 4 % UltraPure agarose 1000 gel (Thermo Fisher Scientific, Waltham MA) with 50-bp molecular weight standards. DNA band sizes were visualized and measured using the gel imaging system and Labworks image acquisition and analysis software v4.6 (UVP BioImaging Systems, Upland, CA). Multiple gametocyte alleles was assessed based on number of bands and differences in band size [45].

For quantification of Pfg377 mRNA, quantitative reverse transcription (RT-qPCR) of Pfg377 was performed as described previously [46]. The standard curve was made with the purified RNA from stage IV and V mature gametocytes by 10-fold dilution series from 10^4^ to 10^0^ gametocytes per µL of blood. All specimens were tested in duplicate; the mean Ct and number of gametocytes were obtained automatically on the Stratagene Mx3005P qPCR system (Agilent Technologies Inc., Santa Clara, CA). The limit of quantification (LOQ) for Pfg377 RT-qPCR was one parasites/µL based on the standard curve established in this laboratory.

### Data sources and statistical analysis

#### Data sources

The 18S-NASBA was used for measuring total parasite load and Pfs25-NASBA was used for measuring gametocyte carriage in both Asembo and Karemo study areas. In addition, Pfg377 RT-PCR and RT-qPCR were performed only on the samples collected in Asembo area for exploring the gametocyte diversity due to funding constraints. Because NASBA uses different approaches for determination of positivity and quantification of parasite density (see NASBA method section above), the positive values for samples with extremely low parasite density could have been below the cut-off of quantitative standard curve, in such positive samples a density was assigned at the midpoint between the cut-off and zero.

#### Data analysis

All data management was performed in SAS 9.3 (SAS Institute Inc., Cary, NC). Population weighted prevalence of parasitaemia and gametocytaemia was calculated via PROC SURVEYFREQ, accounting for field and laboratory sampling scheme and within-household clustering via Taylor series linearization [47]. Model selection and analysis was performed via the MuMIn package [48] and survey packages [49] in R (Version 3.1.3, R Development Core Team, Vienna, Austria) via RStudio (Version 0.98.1073, RStudio Team, Boston, MA).

Multivariable analysis was performed to evaluate participant characteristics associated with parasite or gametocyte presence and density, as well as characteristics associated with presence of multiple gametocyte alleles. Model selection was based on the generalized linear model (GLM) with either Gaussian distribution for continuous outcomes or binomial distribution with a logit link as appropriate for binary outcomes. Best fitting models were selected by lowest Bayesian information criterion (BIC) [50], and full models were then run in the survey package [49] (svyglm and svycontrast) to obtain appropriate odds ratios and confidence intervals for clustered data. Means and confidence intervals (95 % CI) for parasite density were calculated based on the observed marginals.

Association between gametocyte presence and participant characteristics was restricted to individuals who were 18S positive and, therefore, considered infected. Data on parasite and gametocyte density were log_10_ transformed prior to analysis, so estimates are presented as geometric means. Study area was included as a class variable in all models. Where there was no interaction or main effect of study area, overall estimates were presented. Where there was significant impact of study area, area-specific results were presented. Age category of participants was included in all models. The sample collection time was included as a class variable where lowest BIC models included week to account for trends across the sampling timeframe. Anaemia was evaluated as a potential predictor in models for gametocytaemia only. Interactions were not evaluated for gametocyte diversity due to small sample size.

## Results

### Characteristics of study participants

Three smear-positive samples from Karemo were excluded from analysis due to absence of information on fever in previous 24 h, leaving 550 samples from Karemo in this analysis. Thus, characteristics of participants for the study described here were from 996 individuals with complete data. Among 996 participants, 19.8 % of participants reported fever in the past 24 h while 67.5 % of individuals reported sleeping under an ITN the night prior to the survey. Among the 996 participants, 17.8 % had used anti-malarials within the 2 weeks prior to the survey, of which more than 96 % used was AL. Using the haemoglobin cut-off of <11.0 g/dl, 40.3 % of individuals were considered anaemic.

### Parasite and gametocyte profiles by 18S- and Pfs25-NASBA in Asembo and Karemo

**Fig. 1:**
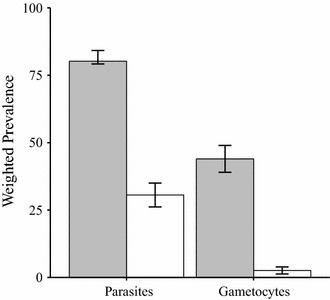
Weighted prevalence of parasitaemia and gametocytaemia measured by 18S-NASBA and Pfs25-NASBA compared to blood smear method for residents of Asembo and Karemo, western Kenya, 2012. It shows weighted parasite prevalence of 80.2 % by 18S-NASBA (grey filled bars) vs 30.6 % by slide smear (*white open bars*) and weighted gametocyte prevalence of 44.0 % by Pfs25-NASBA (*grey filled bars*) vs 2.6 % by slide smear (*white open bars*)

Out of the 996 samples from Asembo and Karemo, 848 (85.1 %) were 18S-NASBA positive (Table 1). Among them, 69.6 % (334/480) of smear-negative samples tested demonstrated submicroscopic infection when measured by 18S-NASBA (Table 1). In Asembo, 18S-NASBA detected 220 (99.6 %) positives from 221 smear-positive samples and identified 135 (60.0 %) positives from 225 smear-negative samples tested (Table 1). In comparison, 18S-NASBA detected 294 (99.7 %) positives from 295 smear-positive samples and 199 (78.0 %) positives from 255 smear-negative samples tested in Karemo (Table 1). Overall, weighted population prevalence of parasitaemia using smear diagnosis was 30.6 % (CI 26.2–35.0 %), relative to a weighted prevalence of 80.2 % (CI 79.2–84.2 %) by 18S-NASBA (Fig. 1). Parasite density was low for many smear-negative samples tested, with 31.5 % (267/848) NASBA positive samples below one parasites/µL of blood. The distribution of parasite density among smear-positive and smear-negative individuals is shown in Additional file 1 , panel A.

**Table 1:** Positivity of *Plasmodium falciparum* parasitaemia and gametocytaemia among samples tested by study areas

Assay	Overall (n = 996)	Asembo		Karemo	
		Smear-positive (n = 221)	Smear-negative (n = 225)	Smear-positive (n = 295)	Smear-negative (n = 255)
18S-NASBA, n (%)	848 (85.1)	220 (99.6)	135 (60)	294 (99.7)	199 (78.0)
Pfs25-NASBA, n (%)	531 (53.3)	177 (80.1)	24 (10.7)	259 (87.8)	71 (27.8)

Overall, 531 out of 996 (53.3 %) samples tested were gametocyte positive by Pfs25-NASBA (Table 1). Weighted population prevalence of gametocytaemia was estimated to be 2.6 % (CI 1.3–3.9 %) by smear diagnosis, relative to 44.0 % (CI 39.0–49.0 %) by Pfs25-NASBA (Fig. 1). Distribution of gametocyte density by Pfs25-NASBA in smear-positive samples had a wide range from 10^−2^ to 10^3^gametocytes/µL, while maximum gametocyte density in smear-negative samples did not exceed 10^2^ gametocytes/µL (Additional file 1, panel B).

### Gametocyte diversity assessed by Pfg377 RT-PCR in Asembo

**Fig. 2:**
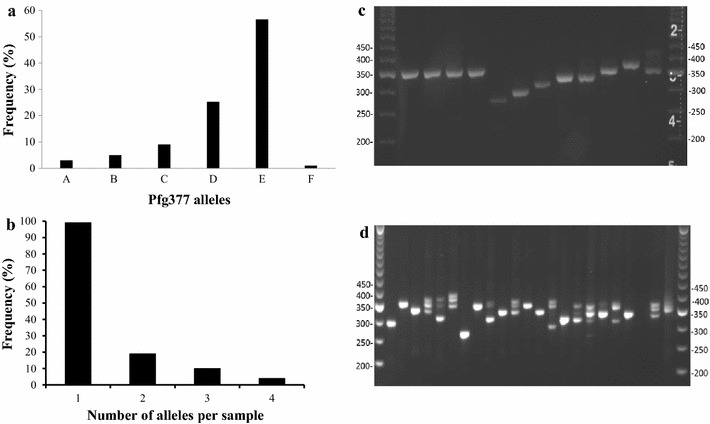
Gametocyte diversity in human blood samples from Asembo, western Kenya (n = 130), 2012. **a** shows the frequency of each single allele of gametocytes detected by Pfg377 RT-PCR. The predominant genotype (56.57 %) is allele E (size 357 bp). **b** shows frequency distribution of Pfg377 alleles per sample. **c** shows each single band on 4 % electrophoresis gel. **d** shows the single or multiple bands of Pfg377 region 3. The gels were run with TrackIt™ 50 bp DNA ladders (Thermo Fisher Scientific, MA)

Pfg377 positivity was detected in 124 of 221 (56.1 %) smear-positive and in 6 of 225 (2.7 %) smear-negative individuals tested in Asembo. Six single gametocyte alleles were identified based on band size (range from 273–378 bps) (Fig. 2a). Out of the 130 Pfg377 positive samples, the single allele infections accounted for 76.2 % (n = 99) and 31 samples were multiple alleles including two alleles (n = 19), three alleles (n = 10) and four alleles (n = 2) within an infection (Fig. 2b). Both single band and multiple bands from the different combination of the six single alleles were seen on agarose gel (Fig. 2c, d).

### Risk factors for the presence of parasitaemia and gametocytaemia

Adjusted odds ratios (aOR) for parasite or gametocyte presence and association with predictors were calculated based on the data of parasite and gametocyte presence in 996 individuals tested (Additional file 2). Overall, age influenced the presence of parasites and gametocytes. Older children (5–15 years old) were more likely to be parasitaemic than individuals >15 years old (OR 2.80, CI 1.46–5.38). Young children (<5 years old) were less likely to be parasitaemic than older children (OR 0.52, CI 0.29–0.95), but not different compared to individuals >15 years old (OR 1.47, CI 0.93–2.31) (Table 2). Similarly, among 18S positive individuals, school aged children were more likely to be gametocytaemic than individuals >15 years old (Pfs25: OR 3.37 CI 2.12–5.36), and young children were less likely to be gametocytaemic than older children (OR 0.42 CI 0.28–0.64). Young children were not different compared to individuals >15 years old in odds for gametocytaemia (OR 1.43, CI 0.95–2.12) (Table 2).

**Table 2:** Adjusted odds ratios (aOR) of parasite and gametocyte presence stratified by risk factor and intervention

aOR	Factor	Overall	Asembo	Karemo
18S	Age: <5 vs >15	1.47 (0.93–2.31)		
	Age: 5-15 vs >15	2.80 (1.46–5.38)^a^		
	Age <5 vs 5–15	0.52 (0.29–0.95)^a^		
	Anaemia vs No Anaemia	NA^b^		
	Fever vs No Fever	1.21 (0.75–1.95)		
	ITN vs No ITN		0.26 (0.10–0.68)^a^	1.08 (0.60–1.93)
	AM vs No AM	1.02 (0.61–1.70)		
Pfs25	Age: <5 vs >15	1.43 (0.95–2.12)		
	Age: 5–15 vs >15	3.37 (2.12–5.36)^a^		
	Age: <5 vs 5–15	0.42 (0.28–0.64)^a^		
	Anaemia vs No Anaemia	2.11 (1.52–2.94)^a^		
	Fever vs No Fever	1.65 (1.11–2.46)^a^		
	ITN vs No ITN	0.78 (0.54–1.14)		
	AM vs No AM	0.32 (0.21–0.50)^a^		

Adjusted odds ratios accounting for multivariate comparisons (18S included age, fever, ITN use, anti-malarial use, study area; Pfs25 included age, fever, ITN use, anti-malarial use, study area, and anaemia). Age was stratified by set age categories (<5, 5–15, >15) in an adjusted analysis. Values were presented by overall for all parameters except for ITN vs No ITN for 18S where there was an interaction by study area and risk factor, reporting by area

^a^Statistically significant

^b^Not analysed because parasitaemia causes anaemia

Anaemia was associated with higher odds of gametocytaemia (OR 2.11, CI 1.52–2.94) (Table 2). Reported fever was also associated with higher odds of gametocytaemia (OR 1.65 CI 1.11–2.46) (Table 2). ITN use was associated with lower odds for parasitaemia in Asembo (OR 0.26, CI 0.1–0.68), but not in Karemo (OR 1.08, CI 0.60–1.93) (Table 2). Anti-malarial (AM) use within the past 2 weeks was associated with lower odds of gametocytaemia by Pfs25 (OR 0.32, CI 0.21–0.50) (Table 2). No other significant predictors were observed in the models predicting parasite or gametocyte presence.

### Risk factors for density of parasitaemia and gametocytaemia

**Fig. 3:**
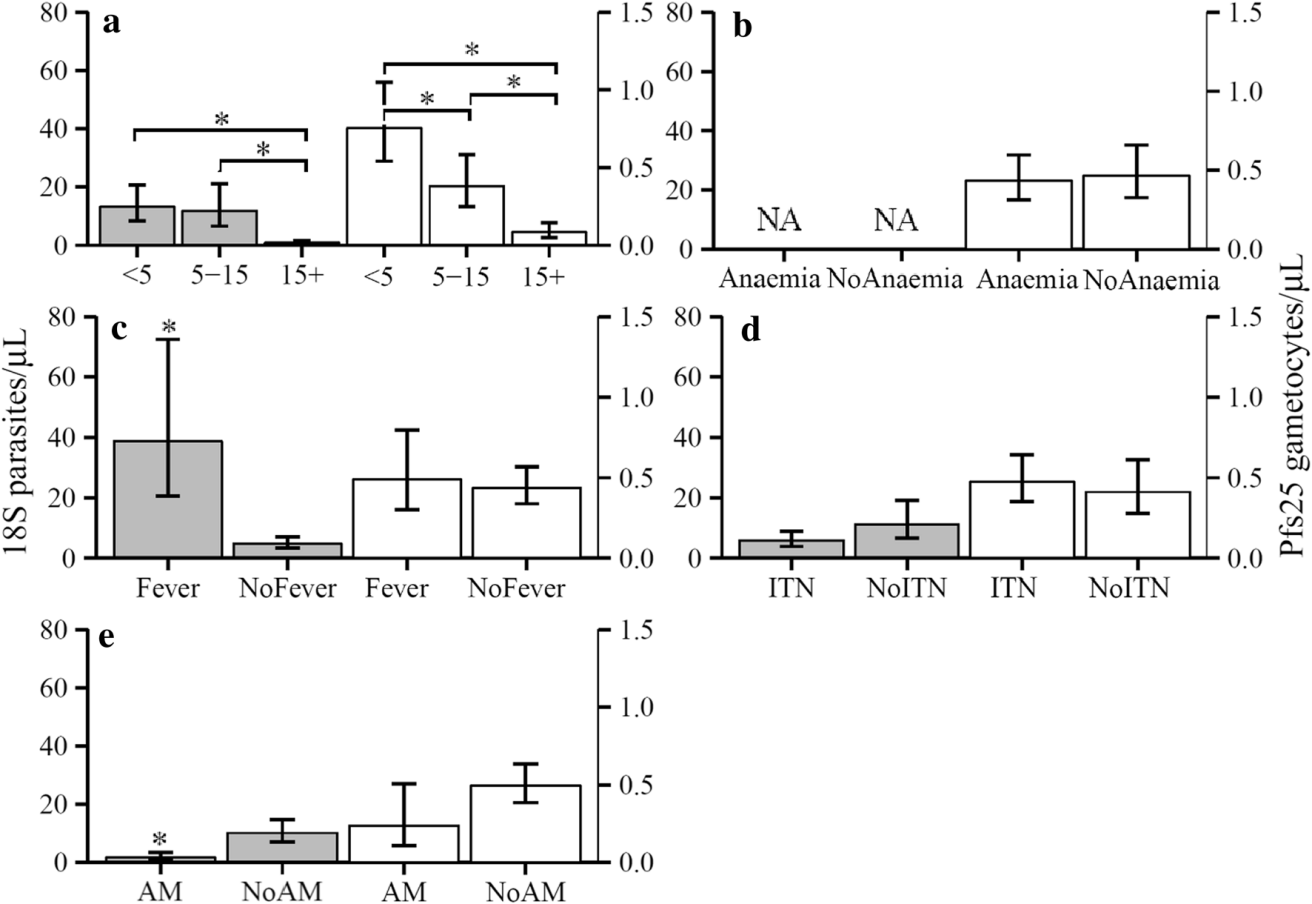
Density of parasites and gametocytes and association with risk factors. Mean densities (± 95 % CI) of parasitaemia by 18S (*grey filled bars*) or gametocytaemia by Pfs25 (*white open bars*) were estimated by GLM, and presented using observed marginals. Left *Y axis* is parasites/µL, while right *Y axis* is gametocytes/µL. The *brackets* indicates the columns compared. *Asterisks* indicates that the column is significantly different (p < 0.05) than the second column with same colour for all *panels*. **a** Density of parasite and gametocyte was lower in individuals >15 years old than young or old children by 18S and Pfs25 (p < 0.001 for all comparisons). **b** Gametocytaemia density did not differ between anaemic and non-anaemic (NoAnaemia) individuals. **c** Parasite density was higher in individuals with fever. **d** There was no associations of ITN use on density of parasitaemia or gametocytaemia. **e** Parasite density was lower among individuals who had received anti-malarials (AM) in the past 2 weeks, but gametocyte density was not significantly different between receiving AM and not receiving AM (NoAM)

The density of parasitaemia and gametocytaemia decreased with age, with individuals >15 years old having the lowest density for both measures (18S and Pfs25 all p < 0.001) (Fig. 3a). Parasite density did not differ between children under 5 and those 5–15 years of age (p = 0.76), but gametocyte density was lower in older children relative to younger children (p = 0.013). Anaemia was not associated with gametocyte density (p = 0.77) (Fig. 3b). Geometric mean parasite density by 18S-NASBA was 7.9 times higher among individuals who had fever in the past 24 h (p < 0.001), but gametocyte density by Pfs25-NASBA was not influenced by fever (p = 0.68) (Fig. 3c). There was no significant association between ITN use and parasite density (p = 0.06) or gametocyte density (p = 0.57) (Fig. 3d). Parasite density was lower among those who had received anti-malarials (AM) in the past 2 weeks (p < 0.001), while gametocyte density was not significantly different between individuals using AM and not using AM (p = 0.067) (Fig. 3e).

### Risk factors for multiplicity of gametocyte infection in Asembo

**Fig. 4:**
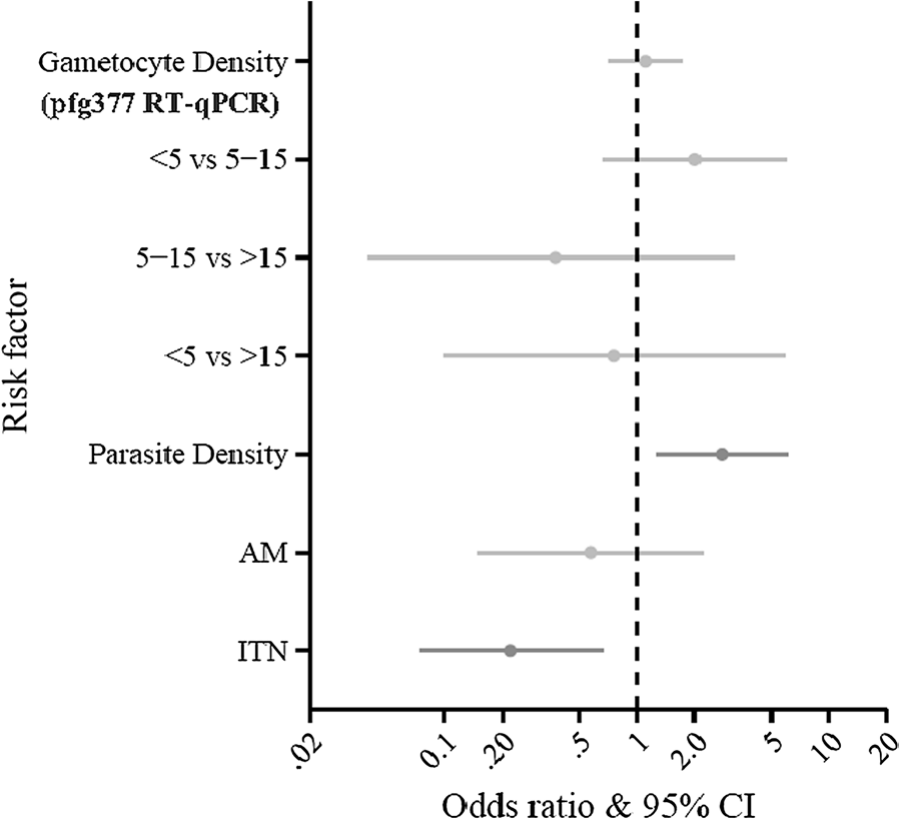
Association of gametocyte diversity with risk factors among Pfg377 positive individuals. Odds ratios for multiple alleles and 95 % confidence intervals. *Grey bars* are non- significant, while *dark bars* indicate significant parameters. Risk factors are on the *Y axes* and odds ratios on the *X axis*. A 10-fold increased parasite density as measured by 18S-NASBA was associated with higher odds of having multiple gametocyte alleles, while sleeping under ITNs was associated with lower odds of having multiple gametocyte alleles

Among Pfg377 positive individuals, age, anaemia and recent anti-malarial treatment did not influence the probability of having multiple gametocyte alleles. However, the odds of having multiple gametocyte alleles were lower in individuals using ITNs than in those not using ITNs (OR 0.22, CI 0.07–0.68, p = 0.0088) (Fig. 4). High parasite density evaluated by 18S-NASBA (each 10-fold increase relative to the mean of 4 × 10^2^ parasites/µL) was associated with higher probability of having multiple gametocyte alleles (OR 2.78, CI 1.25–6.19, p = 0.0126) (Fig. 4), but high gametocyte density by Pfg377 was not associated with multiple gametocyte alleles (p = 0.63).

## Discussion

This study was conducted to estimate submicroscopic infection and gametocyte carriage of *P. falciparum* measured by sensitive RNA-based detection methods and to further assess the risk factors and interventions influencing gametocyte carriage in a community-based cross-sectional survey carried out in western Kenya in 2012 during peak malaria transmission season. Using a subset of samples from the survey, this study revealed an overall high level of submicroscopic parasitaemia (69.6 % of randomly selected smear negative samples) and gametocytaemia (53.3 % of all 18S positive samples tested). When population level estimates were further calculated taking into account of multiple-layer sampling scheme, again a large proportion of infections were submicroscopic. Overall, 80.2 % of the weighted population sample was parasite positive by 18S-NASBA vs 30.6 % by smear diagnosis and 44.0 % of the population was gametocyte positive by Pfs25-NASBA vs 2.6 % by smear diagnosis. Risk factors for gametocyte carriage included being 5–15 years old, anaemia and self-reported fever. The study further showed that the use of anti-malarials within the past 2 weeks was associated with lower odds of gametocytaemia, but not gametocyte density, relative to untreated individuals. Use of ITNs the night before the survey was associated with lower odds of parasitaemia and lower odds of multiple allele gametocyte infection in Asembo.

The overall high level of submicroscopic infection and gametocyte carriage observed in this study was mainly attributed to using the sensitive 18S- and Pfs25-NASBA methods. NASBA is an isothermal nucleic acid amplification reaction that amplifies mRNA in a dsDNA background [51]. The risk of carry-over contamination in NASBA is minimized by the advantage of performing the entire assay in one step closed-tube-format [52]. NASBA is also unique for direct detection of abundant mRNA transcripts, which increases sensitivity compared to DNA-based detection methods [53, 54]. Moreover, gametocyte prevalence assessed by Pfs25-NASBA can be 3.3- to 8-fold higher compared to those assessed by microscopy [9, 10, 55]. Results from the present study were consistent with these previous studies. Although NASBA is highly sensitive in detection of parasites and gametocytes, exact quantification of mRNA by NASBA remains a challenge due to mRNA instability and loss during storage and extraction process, and inherent variability within NASBA amplifications. Therefore, the density measured by NASBA approach was served as a comparative estimate rather than a precise value.

Previous studies have suggested that a high prevalence of submicroscopic gametocytaemia may contribute to malaria transmission [20, 22, 26, 56]. However, there is an argument that transmission is much less likely to occur at submicroscopic gametocyte levels [57]. The impact of extremely low gametocyte density detected by the sensitive Pfs25-NASBA on malaria transmission has not been established, although the value and usefulness of this method has been proven in estimating gametocyte prevalence in previous studies [55, 58] as well as in this cross-sectional survey. Moreover it is known that mere presence of gametocytes does not equate linearly to capacity to infect mosquitoes. And also, this study only measured mature gametocytes by Pfs25 (infectious stage) in blood circulation, not including all immature gametocytes in circulation or sequestered in other tissues. Therefore, it was not a whole picture of gametocyte reservoir in host. In the present study, although there were many more NASBA gametocyte positives from smear-negative individuals (only a subset of smear-negative individuals were selected for this analysis), the majority of NASBA gametocyte positive individuals (80 %) were parasite smear-positive individuals. These results suggested that potential infectious reservoir could be predominantly contributed by individuals with smear-positive infections during peak transmission in the study areas. Together, these results showed a high level of submicroscopic infection and a large submicroscopic gametocyte stage V reservoir at community level, which may contribute to the stagnation in malaria prevalence in western Kenya.

Unlike a clear age-dependent decrease in densities of gametocytaemia (Fig. 3), older children (5–15 year old) had the highest odds for both parasitaemia and gametocytaemia (Table 2). The results from this study were not entirely consistent with previous reports of children <5 years old bearing the highest prevalence of asexual parasites and gametocytes in high endemic settings [17, 19, 59–61]. This new distribution pattern might be a result of the decreased EIR over 9–16 years of interventions in this area. However, the finding that children 5–15 years old were a significant reservoir for potential transmission was consistent with the recent malaria epidemiology in western Kenya [1, 2, 31]. Many factors might account for the unique age patterns for parasitaemia and gametocytaemia observed in the present study. Most likely, the highest odds of parasitaemia and gametocytaemia in older children was attributed to the older children not having been specifically targeted for malaria control programmes and the lower rates of ITN use among this age group [2, 31]. On the other hand, the clear age-dependent decrease of density in gametocytes (Fig. 3a) most likely represented naturally acquired host immunity against both asexual and sexual stage parasites [16, 17, 62, 63]. Whatever reasons for the variations between risk and density for parasitaemia and gametocytaemia, the results from this study indicated that children provided a larger gametocyte reservoir compared to adults in the peak transmission season in western Kenya assuming similar numbers of children and adults in population and similar mosquito bite rates received by children and adults.

Similar to previous studies [11, 64, 65], malaria-associated anaemia was related to the presence of gametocytes in this study. Anaemia is a common condition in *P. falciparum* infection due to the destruction of infected erythrocytes, shortened survival of uninfected erythrocytes and dyserythropoiesis [66–68]. It remains unclear to what extent which mechanisms are involved in the relationship between anaemia and gametocytogenesis. A high proportion of anaemic gametocytaemic individuals could be due to a longer duration of malarial infection [69, 70] or due to reticulocytes [71] and erythropoietin [72] triggering the pathway of gametocytogenesis. In addition, this study showed that high density of parasitaemia and presence of gametocytes measured by sensitive molecular tools were associated with fever. A previous study has reported that children <15 years old in Cameroon with asexual parasitaemia and gametocytes by microscopy have significantly higher prevalence of fever than those without gametocytes [73], while some other studies have reported that gametocyte presence detected by microscopy is negatively associated with fever in *P. falciparum* infection [11, 65]. The relationship between gametocytes and fever is still incompletely understood [74]. But the association of fever with high density parasitaemia and presence of gametocytes observed in this study suggested that the individuals with fever might not get an effective malaria treatment at community level. Collectively, current results indicated that individuals with clinical malaria might be a major gametocyte reservoir in western Kenya.

AL, a highly effective anti-malarial drug with gametocytocidal properties [75], was implemented in western Kenya as a first line treatment drug for uncomplicated malaria in 2006 [27]. In this study population, more than 96 % of anti-malarial drugs reportedly used in the previous 2 weeks were AL. The results showed that, compared to individuals not receiving anti-malarial treatment, AL use within the past 2 weeks was associated with lower odds of gametocytaemia, but not of parasitaemia. In contrast, recent anti-malarial use was significantly associated with lowered parasite density, but not gametocyte density. The different responses to the drugs observed in this study confirmed that AL was more efficiently killing asexual parasites than mature gametocytes in infected individuals and then in turn decreased the chance of developing gametocytaemia in the infected persons. The current results were consistent with previous studies showing that ACT did not decrease proportion of individuals with malaria infection but was associated with a lower rate of gametocyte carriage [26, 29, 76].

This study showed that sleeping under an ITN the night prior to the survey was associated with lower odds of parasitaemia by 18S-NASBA in Asembo, but not in Karemo and also did not influence gametocyte presence among parasitaemic individuals. The different effect of ITN use on the odds of parasitaemia between the two areas could possibly reflect relatively lower level of transmission in Asembo than in Karemo. Entomologic inoculation rates were relatively higher in Karemo in both 2011 and 2012 although the rates were below 10 infectious bites per person per year for both areas (Bayoh, unpublished data). However, these EIR measures were subject to high variation as they were a combined estimate of mosquito densities and sporozoite rates and easily affected by site selection and sampling methods. Parasite positivity as measured by microscopy and by 18S-NASBA in this study was similar between both areas suggesting the differences in EIRs were not substantial. Individual analysis and combined analysis were conducted by study area to explore if there were varying risk factors in these two adjacent areas since they had different ITN implementation time periods (7 years apart). Results from this study confirmed that there were no significant differences in risk factors between Asembo and Karemo except for ITN use on parasitaemia mentioned above.

This study showed that the higher parasite density by 18S NSABA was associated with increased odds of gametocyte diversity. Previous studies have reported that high multiplicity of infection (MOI) is correlated with high malaria transmission level in endemic areas [77–79], and, in particular, is positively correlated with parasite density [78, 80]. Higher parasite density might cause more frequent genetic recombination [80]. Also in this study, individuals reporting use of ITNs were less likely to have high gametocyte diversity. Interestingly, the result from the gametocyte Pfg377 mRNA measurement in this study differed from those of a previous study conducted in the same area. The previous study reported an increase of parasite diversity 5 years after ITN use [81] by assessing a Pfg377 DNA microsatellite locus within a coding region located upstream of region 3 of the Pfg377 gene [82]. It was speculated that the increase in Pfg377 microsatellite diversity at genomic level was a reflection of the parasite population adaptive and survival mechanisms due to reduced transmission [81]. The reason for the discrepancy in results between the gametocyte mRNA transcripts and genomic DNA remains unclear. A recent study in Burkina Faso has shown that only 60 % of all Pfg377 positive samples contains at least one matching genotype between mRNA and genomic DNA [54]. It will be important to further investigate the relationship of gametocyte Pfg377 genetic diversities between stage-specific mRNA transcripts and genomic DNA in the context of malaria prevention and intervention.

This study had a few limitations. First, the sampling strategy of cross-sectional survey in 2012 involved randomly selecting a probability sample of compounds with at least one child under 5 years of age. Therefore, no weighing for geographical density of households was done. Second, self-reported ITN use, fever, recent AL treatment might introduce recall bias among study participants. Third, due to funding constraints, the total number of samples tested for 18S- and Pfs25-NASBA was limited, resulting in full coverage of smear positive samples, but a random selection of smear negative samples from the survey. Because of the multi-level stratified sampling scheme, complex analysis was employed for determination of population prevalence estimates. Both household and laboratory selections were addressed by weighted analysis and all weighted estimates were based on the subset of data selected for laboratory analysis. Although this was a subset of the entire epidemiological dataset with a complex study design, the statistical correction used in this study most likely resulted in representative population estimates of the study area. As well, for resource limited reason, Pfg377 diversity was tested in the samples from the one area with a longer history of ITN interventions for exploration. Although the gametocyte diversity was only examined in this area, the results clearly showed that use of ITNs had a protective role against the gametocyte diversity. The exploratory results could lead to further investigation of gametocyte diversity in relation to sex ratio in context of vector control in future.

## Conclusions

This study showed a large proportion of submicroscopic parasites and gametocytes in western Kenya, which might partially explain stagnation in malaria prevalence and suggests that additional interventions are needed to target the infectious reservoir. As school aged children (5–15 years old) had the highest likelihood for both parasitaemia and gametocytaemia, this age group should be paid more attention in improving the ITN coverage in this area. Gametocyte presence was positively associated with fever and anaemia, indicating patients with clinical malaria or malaria-associated anaemia might be one of major sources for gametocyte reservoir and for potential malaria transmission in western Kenya. Recent AL use reduced parasite density and prevalence of gametocytes, but not the gametocyte density, indicating a limitation of AL in having an impact on transmission reservoir. ITN use played a protective role against parasitaemia and gametocyte diversity in an area with long-standing Asembo area of western Kenya.

## Additional files

10.1186/s12936-016-1482-4 Density distribution of parasites and gametocytes detected by 2 molecular assays. Histograms of parasite and gametocyte densities in age groups as quantified by molecular methods (18S-NASBA in Panel A and Pfs25-NASBA in Panel B). Histogram with filled grey bars represents smear-negative samples, while histogram with open bars represents smear-positive samples. X-axes represent the density of parasite (Panel A) or gametocytes (Panel B) per microliter of blood. Y-axes represents the number of positive individuals by molecular assays.
10.1186/s12936-016-1482-4 Proportion of parasite and gametocyte presence in 996 samples tested by 18S-and Pfs25-NASBA stratified by risk factor and intervention. Proportions are reported in overall and each area, ^$^NA: not analysed because parasitaemia causes anaemia.

## Abbreviations

ITN: insecticide-treated net
ACT: artemisinin-based combination therapy
AL: artemether-lumefantrine
CDC: Centers for Disease Control and Prevention
HDSS: Health and Demographic Surveillance System
DBS: dried blood spot
EIR: entomological inoculation rate
NASBA: nucleic acid sequence based amplification
18S: 18S small subunit ribosomal RNA
Pfs25: 25 kDa ookinete surface antigen
Pfg377: 377 kDa female gametocyte-specific protein
LOQ: limit of quantification
GLM: generalized linear model
BIC: Bayesian information criterion
OR: odds ratio
CI: confidence interval
