# Supplementary figures and images for: Assessment of submicroscopic infections and gametocyte carriage of Plasmodium falciparum during peak malaria transmission season in a community-based cross-sectional survey in western Kenya, 2012
Source: Malar J. 2016 Aug 19;15:421. doi: 10.1186/s12936-016-1482-4 (PMC4992329; doi:10.1186/s12936-016-1482-4)

**Panel A**


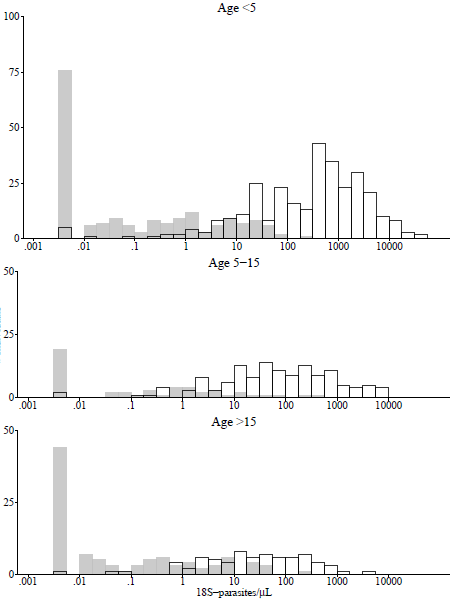


**Panel B**


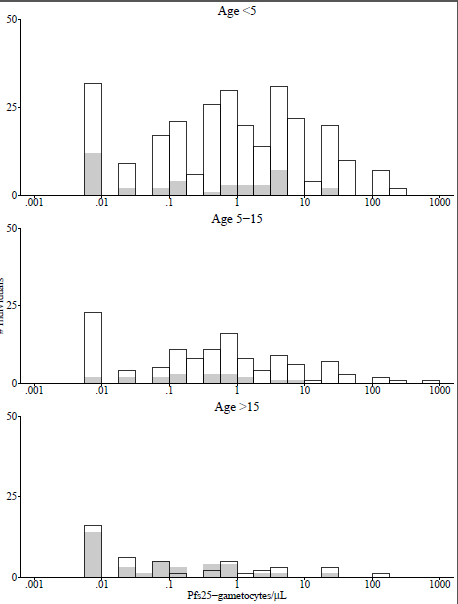

Supplement: Supplementary file 1 — 10.1186/s12936-016-1482-4 Density distribution of parasites and gametocytes detected by 2 molecular assays. Histograms of parasite and gametocyte densities in age groups as quantified by molecular methods (18S-NASBA in Panel A and Pfs25-NASBA in Panel B). Histogram with filled grey bars represents smear-negative samples, while histogram with open bars represents smear-positive samples. X-axes represent the density of parasite (Panel A) or gametocytes (Panel B) per microliter of blood. Y-axes represents the number of positive individuals by molecular assays. [file 12936_2016_1482_MOESM1_ESM.docx]
