# Supplementary material for: Assessment of submicroscopic infections and gametocyte carriage of Plasmodium falciparum during peak malaria transmission season in a community-based cross-sectional survey in western Kenya, 2012
Source: Malar J. 2016 Aug 19;15:421. doi: 10.1186/s12936-016-1482-4 (PMC4992329; doi:10.1186/s12936-016-1482-4)
Supplement: Supplementary file 2 — 10.1186/s12936-016-1482-4 Proportion of parasite and gametocyte presence in 996 samples tested by 18S-and Pfs25-NASBA stratified by risk factor and intervention. Proportions are reported in overall and each area, $NA: not analysed because parasitaemia causes anaemia. [file 12936_2016_1482_MOESM2_ESM.docx]

| **Location** | | | **Freqency-18S** | | | | |  | | | | | | | **Freqency-pfs25** | | |  | | | | |
| --- | --- | --- | --- | --- | --- | --- | --- | --- | --- | --- | --- | --- | --- | --- | --- | --- | --- | --- | --- | --- | --- | --- |
|  | | **Age: <5** | | | | |  | | | | | | **Age: <5** | | | |  | | | | | |
| **Overall** | | 85.4% | | | | |  | | | | | | 53.8% | | | |  | | | | | |
| **Asembo** | | 76.3% | | | | |  | | | | | | 46.5% | | | |  | | | | | |
| **Karemo** | | 91.4% | | | | |  | | | | | | 58.6% | | | |  | | | | | |
|  | | **Age: 5-15** | | | | |  | | | | | | **Age: 5-15** | | | |  | | | | | |
| **Overall** | | 91.5% | | | | |  | | | | | | 69.5% | | | |  | | | | | |
| **Asembo** | | 86.9% | | | | |  | | | | | | 60.7% | | | |  | | | | | |
| **Karemo** | | 96.8% | | | | |  | | | | | | 79.6% | | | |  | | | | | |
|  | | **Age: >15** | | | | |  | | | | | | **Age: >15** | | | |  | | | | | |
| **Overall** | | 78.6% | | | | |  | | | | | | 37.3% | | | |  | | | | | |
| **Asembo** | | 79.3% | | | | |  | | | | | | 27.0% | | | |  | | | | | |
| **Karemo** | | 78.0% | | | | |  | | | | | | 47.7% | | | |  | | | | | |
|  |  | | | |  |  | | | |  | | **Anaemia** | | | | **NoAnaemia** | | |  | |  | |
| **Overall** | | | | NA^$^ | | | | |  | |  | | | 71.0% | | 56.4% | | | |  | | |
| **Asembo** | | | | NA | | | | |  | |  | | | 66.9% | | 51.2% | | | | | |  |
| **Karemo** | | | | NA | | | | |  | |  | | | 73.0% | | 61.1% | | | | | |  |

|  | Fever | NoFever |  | Fever | NoFever |  |
| --- | --- | --- | --- | --- | --- | --- |

| **Overall** | 79.5% | 84.7% |  | | | 59.9% | | 51.7% | |  | | |  |  |
| --- | --- | --- | --- | --- | --- | --- | --- | --- | --- | --- | --- | --- | --- | --- |
| **Asembo** | 79.5% | 73.5% |  | |  | 51.8% | | 43.5% | | |  | | |  |
| **Karemo** | 92.1% | 89.0% |  | |  | 65.8% | | 58.5% | | |  | | |  |
|  | **ITN** | **NoITN** | |  | | | **ITN** | **NoITN** | | | |  |  |  |
| **Overall** | 82.4% | 90.7% |  | | | 48.7% | | 63.0% | |  | | | |  |
| **Asembo** | 75.8% | 90.7% |  | | | 38.3% | | 68.7% | | |  | |  |  |
| **Karemo** | 89.5% | 89.8% |  | | | 59.7% | | 60.4% | | |  | |  |  |
|  | **AM** | **NoAM** |  | | | **AM** | | **NoAM** | | |  | |  |  |
| **Overall** | 85.9% | 85.0% |  | | | 39.0% | | 56.4% |  | | | | |  |
| **Asembo** | 77.3% | 80.0% |  | |  | 25.8% | | 48.4% | | |  | | |  |
| **Karemo** | 91.0% | 89.3% |  | |  | 46.8% | | 63.3% | | |  | |  |  |
